# Long-Term Outcome after Hemithyroidectomy for Papillary Thyroid Cancer: A Comparative Study and Review of the Literature

**DOI:** 10.3390/cancers11010026

**Published:** 2018-12-27

**Authors:** Yossi Geron, Carlos Benbassat, Miriam Shteinshneider, Shlomit Koren, Keren Or, Efrat Markus, Dania Hirsch, Limor Muallem Kalmovich

**Affiliations:** 1 Endocrine Institute, Assaf Harofeh Medical Center, Zerifin 70300, Beer Ya’akov, Israel; carlosb@netvision.net.il (C.B.); stein77m@gmail.com (M.S.); shlomitks@gmail.com (S.K.); or.karen@gmail.com (K.O.); rindefrat@yahoo.com (E.M.); 2 Sackler Faculty of Medicine, Tel-Aviv University, Ramat Aviv 69978, Tel-Aviv, Israel; daniaron@netvision.net.il (D.H.); Limormuallem@gmail.com (L.M.K.); 3 Endocrine Institute, Rabin Medical Center, 39 Jabotinski Street, Petah Tikva 49100, Israel; 4 Head and Neck Surgery Unit, Department of ENT, Assaf Harofeh Medical Center, Zerifin 70300, Beer Ya’akov, Israel

**Keywords:** thyroid, papillary thyroid cancer, hemithyroidectomy, prognosis, recurrence

## Abstract

*Background:* The extent of surgery for differentiated thyroid cancer (DTC) remains a controversial issue. Since a less aggressive approach is becoming more predominant, we aim here to study the short- and long-term outcomes of DTC patients after hemithyroidectomy. *Methods:* From a total of 1252 consecutive papillary thyroid cancer (PTC) patients, 109 treated with hemithyroidectomy and 50 with total thyroidectomy but no I^131^ were included. Persistent or recurrent disease was defined based on histopathology, imaging studies, and thyroglobulin levels. *Results:* Our hemithyroidectomy cohort included females (84.4%), microcarcinomas (81.9%), TNM stage I (95.4%), and a low American Thyroid Association (ATA) recurrence risk (94.5%). At one-year post-treatment, 3.7% had persistent disease (all female, median age 55 years, tumor size 7.5 mm). Recurrent disease was detected in 7.5% of those with excellent response at 1-year. With a follow-up of 8.6 years (1–48), all 109 patients were disease free at last visit, including the 11 patients (10.1%) who received additional treatment. Also, when comparing the hemi- and total thyroidectomy groups no significant differences were found in the rate of persistent and recurrent disease, overall mortality, and disease status at last visit. *Conclusions:* For properly selected low-risk PTC patients, hemithyroidectomy is a safe treatment option with a favorable long-term outcome.

## 1. Introduction

Thyroid cancer is the most prevalent endocrine malignancy worldwide, with an incidence that has nearly tripled over the last two decades [1,2]. Papillary thyroid cancer (PTC), a follicular thyroid cell-derived tumor, accounts for up to 85% of all thyroid cancers [3]. Despite a relatively low mortality rate, disease recurrence/persistence can reach up to 30% [4]. Data on the long-term outcome of patients with well-differentiated thyroid carcinoma (DTC) shows a low rate of disease-specific mortality (DSM), which has been reported in the range of 2–5% when all stages are considered [4]. As most patients who die from DTC are stage IV, and this stage represents a minority of patients, DSM is even lower when considering stages I–III only. Thus, a less aggressive approach has evolved in recent decades, including the option of hemithyroidectomy for tumors up to 4 cm, and even active surveillance for microscopic solitary PTC tumors [5]. Yet, the extent of surgery for thyroid cancer of >1 cm remains a controversial issue. Moreover, studies on disease outcome after hemithyroidectomy in low-risk PTC patients with tumors up to 4 cm are very heterogeneous in methodology and population characteristics.

A landmark study by Bilimoria et al. aroused much of the controversy by showing the statistically significant advantage of total thyroidectomy in comparison to hemithyroidectomy regarding both survival and recurrence [6]. Furthermore, total thyroidectomy was found to be associated with fewer burdens during follow-up [7]. Opponents to this approach argue that hemithyroidectomy is safe and not necessarily associated with a worse outcome if patients are kept under good surveillance [8,9,10,11,12,13]. They also acknowledge that a conservative approach can prevent surgery-related complications, such as hypoparathyroidism and vocal cord paralysis while reducing the need for thyroid hormone replacement [14].

As more data accumulates, the American Thyroid Association (ATA) guidelines shifted toward a more conservative approach [5,14]. While the 2009 guidelines strongly recommended near-total or total thyroidectomy as the initial procedure for tumors larger than 1 cm, the newer 2015 guidelines are not that determined, as evidenced in recommendation 35 B: *“for tumors >1 cm and <4 cm without extrathyroidal extension and without clinical evidence of lymph node metastases can be either total thyroidectomy or hemithyroidectomy”.* Importantly, choosing total thyroidectomy enables radioactive iodine (RAI) ablation, facilitates follow-up, reduces the follow-up burden, and can improve a sense of well-being among survivors [5].

In the present retrospective study, we aimed to contribute more evidence regarding the long-term outcome of PTC patients after hemithyroidectomy, searching for possible prognostic factors and differences with similar stage patients treated with total thyroidectomy.

## 2. Results

From a total of 1252 consecutive papillary thyroid cancer patients treated at the Assaf Harofeh and Rabin Medical Centers, 159 surgically treated patients with a similar TNM stage (109 patients treated with hemithyroidectomy and 50 with total thyroidectomy and no RAI) were considered for this analysis.

Most hemithyroidectomy patients (Table 1) were females (84.4%), had microcarcinomas (81.9%), were at TNM stage I (95.4%), and had a low ATA risk of recurrence (94.5%). In 19/105 patients, the primary tumor was larger than 1 cm, with three of them having tumors in the 2–3 cm range. Multifocality in the resected lobe was observed in 14.7% of our cohort. In addition, 93.5% had no extrathyroidal extension (ETE), and 99.1% had no clinical evidence of lymph node metastases (LNM). RAI for remnant ablation was given to 3.9% of patients. One patient had a 3.5 cm tumor next to the left thyroid lobe which was removed along with the neck mass. The pathology revealed invasive laryngeal chondrosarcoma, and in the thyroid lobe, 4 mm PTC solitary foci was found. One and a half years later, he underwent another neck surgery because of recurrent chondrosarcoma in addition to completion thyroidectomy because of two new thyroid foci of 7 and 5 mm on ultrasound. The pathology this time revealed a 5 mm intrathyroidal PTC foci with a single metastatic PTC lymph node. At this time, he received external-beam radio therapy (EBRT) to the neck because of the chondrosarcoma and 150 millicuries of RAI because of the thyroid disease.

### 2.1. Disease Outcome of PTC Patients after Hemithyroidectomy

The short- and long-term outcomes for the hemithyroidectomy group are shown in Table 2. At one year from the initial treatment, only 3 out of 109 patients (2.7%) had persistent disease; all three were female, the median age was 57 years (range, 44–74 years), and the median tumor size was 9.3 mm (range, 4–13 mm). After completion surgery (100%) and RAI (75%), all of them showed no evidence of disease (NED) at last follow-up visit.

Recurrent disease was detected in 8 (7.5%) out of the 106 patients with excellent response at their 1-year assessment. All recurrences were structural and confined to the neck. The median time from diagnosis to recurrence was 6.9 years (range, 1.5–11.9 years). The additional treatment given to this group, that included completion surgery and RAI, resulted in NED at last visit in all of them.

Disease status reassessed 1-year after the initial treatment revealed a 97.3% rate of excellent response. By the end of the study, with 8.6 years (range, 1–48 years) of follow-up, no evidence of disease was the status for all patients, including the 10.8% to whom additional treatments were given (3 with persistent and 8 with recurrent disease).

### 2.2. Characteristics of Patients with Residual Disease at 1-Year and Last Visit after Hemithyroidectomy

The clinical features of the four patients with incomplete/indeterminate response at the 1-year assessment are shown in Table 3, and for those with recurrent disease during follow-up in Table 4. Patients with persistent/recurrent disease were younger (*p* = ns), more often males (*p* = ns), and had a similar primary tumor size in comparison to the whole hemithyroidectomy group. A comparison between the 11 patients with persistent/recurrent disease and those with an excellent 1-year response and NED throughout the follow-up period failed to reveal any significant difference in their baseline characteristics other than the more extended follow-up period and the RAI therapy given (Table 5).

### 2.3. Disease Outcomes in Low-Risk PTC Patients Treated with Hemithyroidectomy Versus Total Thyroidectomy

The short- and long-term disease outcomes of the hemithyroidectomy group were compared to those treated with total thyroidectomy. The hemithyroidectomy group included only those patients with enough follow-up data that did not receive RAI as initial treatment. The clinical characteristics and disease outcomes for both groups are shown in Table 6. In both groups, the largest tumors were in the range of 2–3 cm (3 patients in the hemithyroidectomy group and 5 patients in the total thyroidectomy group). Overall, there were no differences regarding gender, age at diagnosis, tumor size, focality, and histopathology type. In addition, 95.9% of the patients were defined as TNM stage I in both groups (*p* = ns) and the ATA low-risk of recurrence was also similar (93.9% for hemithyroidectomy, 95.6% for total thyroidectomy, *p* = ns) for both groups. Interestingly, all persistent/recurrent disease patients in the hemithyroidectomy group were found to have structural disease, while all of those with persistent/recurrent disease in the total thyroidectomy group had biochemical-only disease.

In our analysis, we found no significant differences between the hemi- and total thyroidectomy groups for (a) response to treatment at 1-year, (b) rates of recurrent disease, and (c) rates of overall mortality (OM).

## 3. Discussion

The increasing incidence of thyroid cancer is mainly attributed to small papillary thyroid tumors, while the incidence of large tumors and mortality remains unchanged [1,2]. Although surgery is agreed to be the treatment of choice for all DTC patients, the extent of surgery is still a subject of debate, with much of the controversy focused on the long-term disease outcome following hemithyroidectomy [6,8,9,10,11,12,15,16,17,18,19,20,21,22,23,24,25]. Available data on OS (overall survival) and cause-specific survival (CSS), as well as recurrence rates after hemithyroidectomy, yielded conflicting results.

Several studies have been published comparing OS and CSS rates in hemithyroidectomy versus total thyroidectomy, as the initial surgical approach. In a landmark study from Hay et al. [15] on 2444 PTC patients treated at the Mayo Clinic during 1940–1999, hemithyroidectomy was performed in 70% during the first decade, compared to 22% in the second decade, and only 9% after that. DSM and recurrence rates were significantly higher in the hemithyroidectomy group. Interestingly, this study showed that RAI treatment after total thyroidectomy yielded no benefit. Shah et al. [16] compared 73 pairs of DTC patients treated with hemi- and total thyroidectomy. There were no significant differences for local recurrence (7% in both groups) and DSM (11/73 vs. 16/73, hemi- vs. total thyroidectomy). Yet, this study included 23% follicular thyroid cancer (FTC) patients, 33–44% males and 8% M1 patients. Nixon et al. [10] studied 889 patients operated between 1986 and 2005, of whom 637 (83%) had T1 tumors (<2 cm) and 252 (17%) T2 (2–4 cm). Hemi- and total thyroidectomy were performed in 361 and 528 patients, respectively. The groups, however, were significantly different in age, RAI treatment, and non-PTC types. At 99 months of follow-up, there were 64 all-cause deaths, but only one DSM. Despite a single DSM case, the authors went on reporting no differences in CSS rates between groups. Furthermore, OS after stratification by tumor size (<1 cm, 1–2 cm, 2–3 cm, and 3–4 cm) was also similar between groups; however, no data was available on how many of the 80 patients with 3–4 cm tumors were in the hemithyroidectomy group. Importantly, though 0% recurrence rate was reported for the hemithyroidectomy group, 4% needed completion surgery at 6–181 months from diagnosis, with malignancy confirmed in 9 of them (9/361); thus, at variance with the authors reported rate, locoregional recurrences were 2.5% (9/361) vs. 0.8% (5/528), for hemi- and total thyroidectomy, respectively (*p* = 0.123). Adam et al. [12], using the National Cancer Database (NCDB) registry (1998–2006), compared patients with tumors 1–2 cm (60%) versus 2–4 cm (40%) treated with hemithyroidectomy (*n* = 6849) and total thyroidectomy (*n* = 54,926). Again, the groups differed significantly in gender, age, focality, ETE, N1, M1, and RAI treatment; yet, they were similar in tumor size, with 40% in the 2–4 cm range. After multivariate analysis, they found no differences in OS rates between groups. Of note, persistent/recurrent disease was not considered an outcome in this study. Barney et al. [17] reported on DTC patients from the Surveillance, Epidemiology and End Results (SEER) database recorded between 1983 and 2002. The authors found no differences in the rates of OS and CSS between patients treated with hemithyroidectomy (*n* = 3266) versus total/near thyroidectomy (*n* = 15,149). Rates of persistence/recurrence were not considered in this study. Of note, 28% of patients in the hemithyroidectomy group presented had tumors >2 cm compared to 40% in the total thyroidectomy group (*p* < 0.001). Haigh et al. [18] also failed to demonstrate any significant difference in overall mortality (OM), comparing 820 patients after hemithyroidectomy with 4612 after total thyroidectomy. Yet, total thyroidectomy was associated with a higher OM risk but in the low-risk group only.

Interestingly, tumor size was found to be an independent predictor factor for OM in both low- and high-risk patients, regardless of the initial surgical approach. Of note, tumor size was used as a dichotomic variable at 5 cm, and again, persistent/recurrent disease was not among the study outcomes. Despite the apparent flaws described here, these studies were among those considered in the 2015 ATA to recommend hemithyroidectomy as an option in low-risk patients with tumors up to 4 cm.

At variance with the above data, other authors found total thyroidectomy to be a better initial surgical approach. Bilimoria et al. [6] in a study that included 8946 patients after hemithyroidectomy and 43,227 after total thyroidectomy from the NCDB found higher rates of recurrence (*p* = 0.04) and lower rates of survival (*p* = 0.009) among low-risk patients with tumors >1 cm that were treated with hemithyroidectomy compared to total thyroidectomy. Similar results were seen when the 1–2 cm tumors were analyzed in separate. In our study, persistent disease 1-year after a hemithyroidectomy was present in only 2.7% of patients and recurrent disease after excellent response in another 7.3% patients. Yet, with a median follow-up of 8.6 years, and after additional treatment they all achieved NED at last follow-up visit. Mazzaferri et al. [19] reported on 825 patients treated with total or near-total thyroidectomy versus 300 treated with hemithyroidectomy only and found higher rates of OM in the latter group. Similar findings were observed by Loh et al. [20] who reported a 2.2 hazard ratio (HR) for DSM and 2.5 HR for recurrence in DTC patients treated with hemithyroidectomy (*n* = 133) compared to total thyroidectomy (*n* = 551). In a metanalysis by Macedo et al. [21], including 6 studies conducted between 2002 and 2013, for a total of 901 patients treated with hemithyroidectomy and 2134 with total thyroidectomy, the authors found 1.5 more recurrences and 1.7 more DSM in the hemithyroidectomy group (62/801 and 8/801 vs. 93/2134 and 8/2134, respectively). In a second metanalysis by Guo et al. [22] including 7 studies conducted between 2004 and 2013 for a total of 618 hemithyroidectomies and 1998 total thyroidectomies, they found 2.3 times more recurrences in the hemithyroidectomy group.

Several well-recognized predictive factors for recurrence are taken into consideration when deciding upon the extent of surgery, among which are tumor size, ETE, and clinically evident LNM [5]. Though previous studies showed ETE and multifocality as predictors of recurrent disease following hemithyroidectomy [5,8], we failed to identify any predictors of a worse outcome in our group. Regarding the extent of surgery, some studies found hemithyroidectomy to be associated with higher recurrence rates [6,23,24], while others like us did not [10,11,12]. Nevertheless, our cohort was too small to reach significant conclusions.

Several other authors have investigated the risk of recurrence after hemithyroidectomy. Matsuzu et al. [11] studied 1088 PTC patients with a median tumor size of 2.2 cm, all of whom were treated with hemithyroidectomy, and found recurrent disease in only 52 of them (4.6%). Interestingly, while half of the tumors were ≥4 cm, the authors used 4 cm as a dichotomic variable; thus, the outcome for tumors in the 2–3 and 3–4 cm range was not analyzed. Huang et al. [25] followed 123 unilateral multifocal PTC patients after hemithyroidectomy and found recurrent disease in 11.4% of them. Similarly, Li et al. [8] reported a 4.6% recurrence rate in 129 patients after hemithyroidectomy, and Gershinsky et al. [26] reported completion surgery in 10% of low-risk patients. Kwon et al. [27], studying 688 matched microscopic PTC patients, found recurrent disease in 3.8% and 1.6% after hemi- and total thyroidectomy, respectively (*p* > 0.01). The time to recurrence in the hemithyroidectomy group was 4.4 years from diagnosis and, as in our study, they all achieved NED after completion thyroidectomy. Vaisman et al. [28] studied 289 patients, of whom 72 underwent hemithyroidectomy and found recurrent disease in only 3 of them (4.2%), all of whom achieved NED after completion surgery. Kim et al. [16] reported no difference in recurrence rates for 1 to 4 cm PTC tumors (85% < 2 cm) treated with hemi- versus total thyroidectomy (6.1% vs. 5.7%, respectively).

Altogether, the above data demonstrate that salvage surgery after hemithyroidectomy, with or without RAI, appears to be effective in removing residual disease, avoiding new recurrences or increased mortality throughout 10 years of follow-up. It also indicates that adherence to ATA risk stratification and proper patient selection before surgery can minimize the need for completion surgery. The 2015 ATA guidelines embraced most of the above studies to finally recommend hemithyroidectomy as an acceptable first-line treatment for low-risk patients with tumors up to 4 cm [5]. Yet, since most studies on hemithyroidectomy included patients with tumors <2 cm or microscopic PTC [23,27,28,29,30] and many of them focused on survival rather than recurrence; concern exists on whether the evidence is sufficient to support hemithyroidectomy as initial treatment in low-risk patient with tumors up to 4 cm. Moreover, an Israeli survey [31] conducted just after the 2015 ATA guidelines were released found that less than 50% of responders would agree to perform hemithyroidectomy for a unilateral 4 cm low-risk PTC tumor. The proportion of patients undergoing hemithyroidectomy for 2–4 cm unilateral tumors was 42% in Matsuzu’s study [11], 40% in Adam’s study [12], 33% in Bilimoria’s research [6], and less than 18% in most other studies [17,32]. Furthermore, the proportions of tumors in the 2–3 and 3–4 cm range, as well as their respective long-term outcomes have not been fully investigated.

The current controversy on the extent of surgery probably rises from the heterogeneity in aims and design among studies. The varied aims included were: survival [6,12,15,16,17,18,19,20], multifocality [8,25,30,33], need for hormone replacement [7,34], contralateral nodules or completion for indeterminate cytology [28,33,35,36], among others. Nevertheless, by using propensity score matching, two recent studies found that the extension of surgery has no impact on disease outcomes. Lee et al. [29] compared 506 pairs of PTC patients treated with hemithyroidectomy or total thyroidectomy. The recurrence rates at 5, 10, and 20 years were non-statistically different (1.9%, 4.3%, and 6.5% vs. 2.3%, 4.1%, and 5.9%, hemi- vs. total thyroidectomy, respectively), and no differences were seen for OS rates as well. Kuba et al. [37] reported similar findings on 33 pairs of macroscopic PTC patients. The results of the above studies are in accordance with those reported by us.

Postoperative follow-up using I^131^ whole-body scan (WBS) and thyroglobulin (Tg) levels are among the main reasons favoring total thyroidectomy. However, high-resolution neck ultrasound appears more accurate for the detection of residual/recurrent disease than WBS [38]. Thus, a possible negative impact of a hemithyroidectomy on survival can be avoided with careful surveillance [10,11,25,26,27,39]. While total thyroidectomy enables the addition of RAI treatment, a large study using the NCDB database [40] showed postoperative RAI ablation was given to only 56% patients, suggesting that preparation for RAI ablation was not a factor on deciding the extent of surgery. Accordingly, the new ATA guidelines do not recommend routine RAI treatment for micro, small, or even minimal ETE tumors [5]. Similarly, the yield of the Tg measurement for detecting recurrence after hemithyroidectomy is quite limited [41,42] and the ATA guidelines (recommendation 62.c) state that its utility in low-to-intermediate-risk patients who achieve an excellent response to initial treatment is not well established [5]. Finally, clinicians should keep in mind that more extensive surgeries could result in more related complications [14].

Our study has several limitations. Firstly, its retrospective design inherently carried missing information; thus, we provided data for each variable, both as percentages and number of patients with available data. Secondly, the small sample in our study precludes us from reaching substantial and significant conclusions. Thirdly, propensity score analysis would have been very appropriate for the comparison between groups; however, for the propensity analysis, the number of matched pairs needed required a study sample big enough to make it feasible. Fourthly, in the hemithyroidectomy group, follow-up was almost twice in patients with recurrence. The difference in follow-up could have biased our results; yet, the long enough 8 years median follow-up in the non-persistent group, and the fact that recurrences were diagnosed earlier, argues against a possible bias. Finally, surgical complications were only partially recorded in our cohort, thus not included in our analysis. Yet, other authors did address this issue and found hemithyroidectomy to be associated with fewer complications [14].

Most of our patients were TNM stage I (95.4%), and with low ATA risk for recurrence (94.5%); therefore, our conclusions should only be applied to similar patients. Nevertheless, as the trend toward a more conservative approach in low-risk patient continues, the existing cumulative data, including the present study, support the concept that for this subgroup of patients, total thyroidectomy and RAI treatment offer little or no benefit. Whether the same can be said for low-risk patients having tumors up to 4 cm, as suggested in the 2015 ATA guidelines, needs to be confirmed by future large and long-term studies.

## 4. Materials and Methods

### 4.1. Patients

The Assaf Harofeh and Rabin Medical Centers’ thyroid cancer registries were searched for all patients with differentiated PTC who have retrospectively registered since 2005. The ethical board approved this study at both institutions (Assaf Harofeh Medical Center ethics code: 0072-16-ASF, permission date: 28 May 2018 throw 17 July 2019; Rabin Medical Center ethics code: 0403-12-RMC, permission date: 6 August 2017 throw 29 October 2018). In accordance with Helsinki regulations regarding clinical studies based solely on the review of charts, informed consent was waived by the ethics committees at both institutions, requiring only for individuals to be given a serial number and be kept in anonymity. Medical charts were reviewed, and patients with a proven diagnosis of papillary thyroid cancer initially treated with hemithyroidectomy were included, along with a group of unilateral PTC patients for whom total thyroidectomy without RAI therapy was indicated based on their low-risk preoperative assessment. In order to avoid confounding effects and selection bias, the following exclusion criteria were applied: (1) Histopathology types other than PTC follicular and classical variants; (2) insufficient data for analysis; (3) palliative hemithyroidectomy for widely invasive tumors; (4) total thyroidectomy for extensive locoregional and/or metastatic disease; (5) total thyroidectomy for proved bilateral disease; (6) RAI following surgery. In both groups, unilateral multifocal disease was not excluded. In our retrospective study, these selection criteria partially compensate the lack of an exact matching design as it allows to compare patients with a similar stage and no additional treatment given at diagnosis.

The following clinical variables were collected from the registry: patient age and sex, type and time of surgery, histopathology findings, the extent of primary disease, extrathyroidal extensions, TNM staging, and Tg levels, as well as the number and type of additional treatments when needed. All patients were monitored by standard methods, including clinical examination, thyroid function tests, a neck sonogram, radioiodine scan, serum Tg, and anti-Tg antibodies. Some patients also underwent other radiological tests, as required, including computed tomography, positron emission tomography-computed tomography, magnetic resonance imaging, and a bone scan. The TNM staging system was based on the American Joint Committee on Cancer/International Union for Cancer Control 7th edition, and the risk of recurrence was defined by the 2015 ATA thyroid cancer guidelines [5].

Persistent disease was defined based on histopathology findings (fine needle aspiration preoperatively, pathology postoperatively), imaging studies (scintigraphy, CT, MRI, ultrasound), and Tg levels within one year from initial therapy. Recurrent disease was considered for patients showing no evidence of disease at 1-year from initial treatment and for whom the above findings developed during the follow-up. The definition of biochemical, structural, and indeterminate recurrence was the one adopted by the 2015 ATA guidelines [5]. Disease-free status or remission was defined as the absence of structural disease combined with undetectable suppressed Tg and/or at least one stimulated thyroglobulin measurement ≤ 2 ng/mL in the absence of anti-Tg antibodies.

Quality control of the registry data was performed by data verification on several occasions. The levels of thyroid-stimulating hormone, free T4, Tg, and anti-Tg antibodies were measured by a chemiluminescence assay (Immulite, 2000; Diagnostic Products Corp, Los Angeles, CA, USA). For the Tg assay, the functional sensitivity was 0.9 ng/mL, and the analytical sensitivity was 0.2 ng/mL.

### 4.2. Statistical Analysis

Numerical data were expressed as mean and standard deviation, median, and range, and categorical data as number and percent. Groups were compared using an independent Student’s *t*-test for numerical variables and a chi-square test or Fisher’s exact test for categorical variables. A *p* value of <0.05 was statistically significant. SPSS software was used for all statistical analyses (IBM SPSS statistics for windows, version 24, IBM corp., Armonk, NY, USA, 2016).

## 5. Conclusions

We conclude that total thyroidectomy as the first-choice treatment for low-risk DTC patients with unilateral solitary PTC tumors has no significant benefits compared to hemithyroidectomy and the low-risk patients may undergo hemithyroidectomy without jeopardizing the cure rates.

## Figures and Tables

**Table 1:** Baseline characteristics and disease outcome in 109 papillary thyroid cancer (PTC) patients treated with hemithyroidectomy.

Baseline Characteristics	No. of Patients
Female	92/109 (84.4%)
Age at diagnosis, Mean ± SD	50.1 ± 13.3
Irradiation exposure	6/88 (6.8%)
Family History	6/88 (6.8%)
Hypothyroidism at diagnosis	17/80 (21.3%)
Neck Dissection performed	2/109 (1.8%)
Multifocal Disease	16/109 (14.7%)
PTC Classical Variant	87/109 (79.8%)
Follicular Variant	22/109 (20.2%)
Vascular invasion	2/105 (1.9%)
Extrathyroidal Extension	7/108 (6.5%)
Microcarcinoma (<10 mm)	86/105 (81.9%)
Tumor size, median (range), mm	8 (1–30)
Tumor stage 1	98/106 (92.5%)
stage 2	3/106 (2.8%)
stage 3	5/106 (4.7%)
stage 4	0/106 (0%)
Lymph node metastases at diagnosis	1/106 (0.9%)
Distant metastases at diagnosis	0/109 (0%)
TNM stage I	103/108 (95.4%)
stage II	0/108 (0%)
stage III	5/108 (4.6%)
stage IV	0/108 (0%)
ATA Low recurrence risk	103/109 (94.5%)
Intermediate risk	6/109 (5.5%)
High risk	0/109 (0%)
I^131^ treatment given	4/103 (3.9%)

ATA: American Thyroid Association.

**Table 2:** Short- and long-term outcomes of 109 papillary thyroid cancer patients after hemithyroidectomy.

Response to Treatment at 1-year	No. of Patients
Excellent	106 (97.3%)
Incomplete (all structural)	2 (1.8%)
Indeterminate	1 (0.9%)
Recurrence (all regional)	8/106 (7.5%)
Biochemical	0/106 (0%)
Structural	8/106 (7.5%)
Retreatment, all	11/102 (10.8%)
Additional I131 therapy	9/101 (8.9%)
Reoperation	11/109 (10.1%)
Persistent disease at last visit	0/107 (0%)
Mortality all	7/109 (6.4%)
Related	0/109 (0%)
Unrelated	5/109 (4.6%)
Unknown	2/109 (1.8%)
Follow-up, yrs., median (range)	8.6 (1–48)

**Table 3:** Baseline characteristics of papillary thyroid cancer (PTC) patients with incomplete response to initial treatment at 1-year reassessment.

Case	Age	Gender	Size (mm)	PTC Type	ETE	Focality	N1	M1	1-Year Response to Treatment
1	44	Female	13	PTC	no	Unifocal	-	-	Structural Persistence
2	53	Female	11	PTC	no	Unifocal	-	-	Structural Persistence
3	74	Female	4	PTC	no	Unifocal	-	-	Indeterminate

ETE, extrathyroidal extension; N1, lymph node metastasis; M1, distant metastasis.

**Table 4:** Baseline characteristics and disease outcome of papillary thyroid cancer (PTC) patients with excellent response at 1-year and disease recurrence during follow-up.

Case	Age Dx	Gender	Size (mm)	PTC Type	ETE	Focality	N1	M1	Follow Up Recurrence	Additional Treatments	Last Visit		F/U Years
											Status	Death	
1	55	M	1	PTC	No	Unifocal	-	-	Structural	ReOp, I^131^	NED	No	15.9
2	46	F	8	PTC FV	No	Multifocal	-	-	Structural	ReOp, I^131^	NED	No	20.5
3	39	F	15	PTC FV	No	Unifocal	-	-	Structural	ReOp, I^131^	NED	No	30.7
4	74	F	16	PTC	No	Unifocal	-	-	Structural	ReOp	NED	No	10.3
5	27	F	10	PTC FV	No	Multifocal	-	-	Structural	ReOp, I^131^	NED	No	20.0
6	46	F	10	PTC	No	Unifocal	Yes	-	Structural	ReOp, I^131^	NED	No	19.1
7	57	M	4	PTC	No	Unifocal	-	-	Structural	ReOp, I^131^	NED	No	8.2
8	60	M	10	PTC	No	Unifocal	-	-	Structural	ReOp, I^131^	NED	No	18.3

ETE, extrathyroidal extension; FV, follicular variant; Dx, diagnosis; N1, lymph node metastasis at diagnosis; M1, distant metastasis a diagnosis; ReOp, reoperation; I^131^, radioactive iodine; NED, non-evidence of disease; F/U, follow-up.

**Table 5:** Comparison of baseline characteristics between papillary thyroid cancer (PTC) patients with and without persistent/recurrent disease.

Baseline Characteristics	Persistent/Recurrent Disease		*p*-Value
	No = 98	Yes = 11	
Female	85.7%	72.7%	ns
Age at diagnosis, Mean ± SD	49.9 ± 13.3	52.3 ± 14.1	ns
Irradiation exposure	5/77 (6.5)	1/11 (9.1)	ns
Family History	5/79 (6.3)	1/9 (11.1)	ns
Hypothyroidism at diagnosis	16/72 (22.2)	1/8 (12.5)	ns
Neck Dissection performed	1/98 (1)	1/11 (9.1)	ns
Multifocal Disease	14/98 (14.3)	2/11 (18.2)	ns
PTC Classical Variant	80.6%	72.7%	ns
Follicular Variant	19.4%	27.3%	-
Vascular invasion	2/94 (2.1)	0/11 (0)	ns
Extrathyroidal Extension	7/97 (7.2)	0/11 (0)	ns
Microcarcinoma	79/94 (84)	7/11 (63.6)	ns
Tumor size, median (IQR), mm	7 (5–10)	10 (4–13)	ns
Tumor stage 1	87/95 (91.6)	11/11 (100)	ns
stage 2	3/95 (3.2)	0/11 (0)	-
stage 3	5/95 (5.3)	0/11 (0)	-
stage 4	0/95 (0)	0/11 (0)	-
Lymph node metastases at diagnosis	0	1/11(9.1)	ns
Distant Metastases at diagnosis	0	0	ns
TNM stage I	93/97 (95.9)	10/11 (90.9)	ns
stage II	0	0	-
stage III	4/97 (4.1)	1/11 (9.1)	-
stage IV	0	0	-
ATA Low recurrence risk	92/98 (93.9)	11/11 (100)	ns
Intermediate risk	6/98 (6.1)	0	-
High risk	0	0	-
I^131^ treatment given	2/93 (2.2)	2/10 (20)	0.046
Follow-up, yrs., median (IQR)	8.1 (3.7–13.5)	18.1 (10.3–20.0)	0.024

Proportions are expressed as n (%); ATA, American Thyroid Association; IQR, interquartile range; ns: non-significant.

**Table 6:** Baseline characteristics and disease outcome of unilateral papillary thyroid cancer (PTC) patients treated with hemithyroidectomy and total thyroidectomy with no RAI as initial treatment.

Baseline Characteristics	Hemithyroidectomy (*n* = 99)	Total Thyroidectomy (*n* = 50)	*p*-Value
Female	83 (83.8)	40 (80)	ns
Age at diagnosis, Mean ± SD	49.9 ± 13.2	51.8 ± 16.8	ns
Tumor size, median (range), mm	8 (1–30)	6 (1–30)	ns
Multifocal Disease	13 (13.1)	7 (14)	ns
PTC Classical Variant	78 (78.8)	41 (82)	ns
Follicular Variant	21 (21.2)	9 (18)	-
TNM stage I–II	94 (95.9)	47 (95.9)	ns
stage III–IV	4 (4.1)	2 (4.1)	-
ATA low recurrence risk	93 (93.9)	44 (95.6)	ns
Intermediate risk	6 (6.1)	1 (2.2)	-
High risk	0	1 (2.2)	-
Excellent response at 1-year	96 (97)	49 (98)	ns
Recurrence	6 (6.1) *	1 (2.3) ^†^	ns
All-cause mortality	6 (6.1)	7 (14)	ns
Follow-up, yrs., median (IQR)	8.3 (3.8–14.8)	7.1 (3.3–18.7)	ns

Proportions are expressed as n (%); ATA, American Thyroid Association; IQR, interquartile range; RAI, radioactive iodine. * Recurrences in the hemithyroidectomy group were all structural; † Recurrences in the total thyroidectomy group were all biochemical; ns: non-significant.

## Abbreviations

ATA	American Thyroid Association
CSS	Cause-specific survival
DSM	Disease-specific mortality
DTC	Differentiated thyroid cancer
EBRT	External-beam radio therapy
ETE	Extrathyroidal extension
FTC	Follicular thyroid cancer
HR	Hazard ratio
LNM	Lymph node metastases
NCDB	National Cancer Database
NED	No evidence of disease
OM	Overall mortality
OS	Overall survival
PTC	Papillary thyroid cancer
PTCFV	Papillary thyroid cancer follicular variant
RAI	Radioactive iodine
SEER	Surveillance, Epidemiology and End Results
Tg	Thyroglobulin
WBS	Whole-body scan
